# Incomplete Kawasaki disease complicated by coronary aneurysm: a case report

**DOI:** 10.1093/ehjcr/ytaf056

**Published:** 2025-02-19

**Authors:** Mariana Caetano Coelho, Fernando Ferreira, Alexandra Castelo, João Reis, Rui Cruz Ferreira

**Affiliations:** Department of Cardiology, Hospital Santa Marta, Rua de Santa Marta 50, Lisbon 1169-024, Portugal; Department of Cardiology, Hospital Santa Marta, Rua de Santa Marta 50, Lisbon 1169-024, Portugal; Department of Cardiology, Hospital Santa Marta, Rua de Santa Marta 50, Lisbon 1169-024, Portugal; Department of Cardiology, Hospital Santa Marta, Rua de Santa Marta 50, Lisbon 1169-024, Portugal; Department of Cardiology, Hospital Santa Marta, Rua de Santa Marta 50, Lisbon 1169-024, Portugal

**Keywords:** Incomplete Kawasaki disease, Coronary artery aneurysms, Myocardial infarction, Coronary artery bypass grafting, Case report

## Abstract

**Background:**

Kawasaki disease is a vasculitis affecting medium-sized vessels, particularly coronary arteries, and is a leading cause of acquired heart disease in children. It is diagnosed clinically based on prolonged fever and specific symptoms. We present a case of incomplete Kawasaki disease, highlighting how it can result in delayed diagnosis and elevate the risk of coronary artery aneurysms.

**Case summary:**

A 31-year-old patient with an unrecognized history of Kawasaki disease presented to the emergency department with symptoms of angina. Imaging revealed significant coronary artery aneurysms, and he successfully underwent coronary artery bypass grafting.

**Discussion:**

Early recognition and treatment of Kawasaki disease are crucial to prevent serious complications like coronary artery aneurysms.

**Learning points**

- Incomplete Kawasaki disease can be challenging to diagnose, increasing the risk of coronary artery aneurysms due to potential delays in treatment with intravenous immunoglobulin.
- Coronary artery aneurysms are often found incidentally but can lead to serious complications. Treatment options, including surgical, percutaneous, and medical interventions, are available, though the optimal approach remains uncertain.
- Improved detection of coronary artery aneurysms through advanced imaging technologies underscores the need for clear, evidence-based management strategies.

## Introduction

Kawasaki disease (KD) is a medium-sized vessel vasculitis that primarily affects the coronary arteries, often presenting as an acute and self-limiting condition.^1^ Kawasaki disease holds the position as the second most frequently occurring vasculitis during childhood and the leading cause of acquired heart disease in developed countries.^2^ The diagnosis of KD is clinical and is based on typical symptoms such as fever lasting for more than 5 days and showing at least four of the five principal clinical features as polymorphous rash, mucosal changes like dry, cracked lips and a ‘strawberry’ tongue, erythema, swelling, desquamation of the palms and soles, bilateral non-purulent conjunctivitis, and cervical lymphadenopathy (*Table 1*).^1^ Incomplete KD (IKD) is diagnosed in cases of unexplained fever where patients do not meet the full diagnostic criteria (*Table 2*). Adolescents with IKD are at higher risk for coronary artery aneurysms (CAAs) due to potential delays in treatment with aspirin and intravenous immune globulin (IVIG), which reduces coronary involvement from ∼25% to <4%.^3^ CAA, defined as coronary artery dilation exceeding 50% of the reference vessel diameter, is rare, with atherosclerosis being the primary cause in adults and KD in children.^4^ Although treatment options include surgical, percutaneous, and medical interventions, the optimal approach is still debated. With advancements in invasive coronary angiography (ICA), high-resolution computed tomography (CT), and magnetic resonance imaging, the diagnosis of CAA is expected to rise, underscoring the need for evidence-based management strategies for this rare condition.

**Table 1:** Kawasaki disease: diagnostic criteria

Fever lasting 5 days, along with four of the five criteria (Kawasaki disease can be diagnosed with fewer than four of these features if coronary artery abnormalities are detected)
Conjunctival injection (bilateral, non-exudative, painless, limbic sparing)
Rash (erythematous polymorphous rash within the first days, involving the trunk and extremities, most often presenting as maculopapular, erythema multiforme-like, or scarlatiniform)
Oral changes (strawberry tongue)
Extremity changes (hyperaemia and painful oedema of the hands and feet progress to desquamation starting in the second week of illness)
Lymphadenopathy (cervical lymphadenopathy, most commonly unilateral and tender, with at least one node larger than 1.5 cm, is a less common feature and typically seen in older children)

**Table 2:** Key indicators for considering incomplete Kawasaki disease diagnosis

Fever lasting at least 5 days and:
2 or 3 of the principal clinical features;
Irritability without other explanation; and
Prolonged fever and unexplained aseptic meningitis, shock, or cervical adenitis not responsive to oral antibiotics.

Here, we present a case of IKD disease in a 14-year-old boy, who initially exhibited symptoms of fever, painful cervical lymphadenopathy, and conjunctivitis. These symptoms were later complicated by the development of a coronary artery aneurysm at the age of 31.

## Summary figure

Timeline of clinical events illustrating the progression of incomplete Kawasaki disease into coronary aneurysm. CABG, coronary artery bypass graft surgery.

**Figure:**
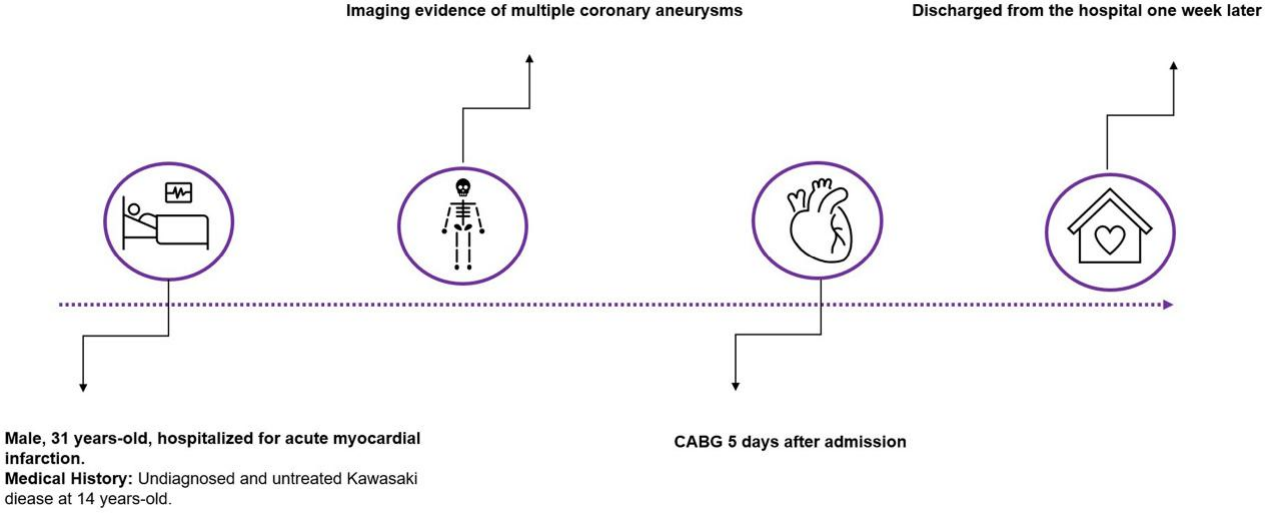


## Clinical case

A 31-year-old male patient, presenting with typical angina symptoms upon exertion, sought care at the emergency department. Notably, at the age of 14, he experienced an episode marked by persistent fever, painful cervical lymphadenopathy, and conjunctivitis. Despite elevated inflammatory markers and extensive diagnostic workups, no definitive diagnosis was made. He was treated for inflammatory pericardial effusion, which required drainage, and was given empirical antibiotics. After three weeks of supportive care and further investigation into potential Still’s disease, he was discharged on prednisolone. Over the years, he remained stable without further symptoms or complications.

Upon admission, the physical examination was unremarkable, vital signs were blood pressure of 135/82 mmHg, pulse of 82 beats/min, respirations of 14 breaths/min, oxygen saturation (O2 sat) of 100%, and temperature of 37.1°C. Cardiac and pulmonary auscultation revealed no pathological findings, and the extremities were well-perfused without oedema.

The electrocardiogram showed dynamic changes, including inverted T waves in lead aVF and leads V5–V6. Blood tests revealed elevated high-sensitivity cardiac troponin T, peaking at 657 ng/L (normal < 34 ng/L). No other abnormalities were found in the blood tests, including a normal BNP (B-type natriuretic peptide) level. Echocardiography indicated preserved systolic function with no segmental alterations and no significant changes, including aortic dissection.

**Figure 1:**
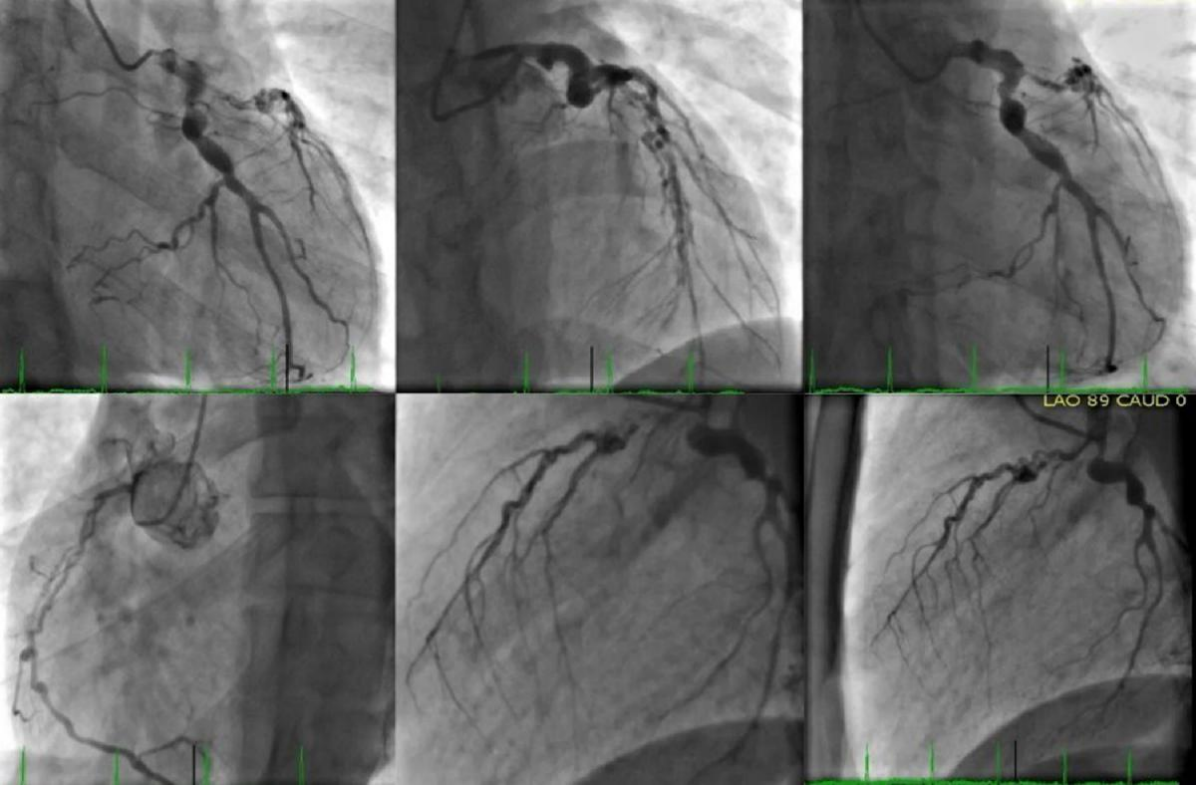
Invasive coronary angiography—aneurysmal disease of the left main trunk and chronic total occlusion of the left anterior descending artery. A medial aneurysm of the left circumflex artery with 70% occlusion.

The patient underwent ICA (*Figure 1*) that revealed left main trunk aneurysmal disease and chronic total occlusion (CTO) of the left anterior descending (LAD) artery, with a medial aneurysm of the left circumflex (LCx) artery 70% occluded, and a CTO of the mid right coronary artery (RCA) with several collaterals.

**Figure 2:**
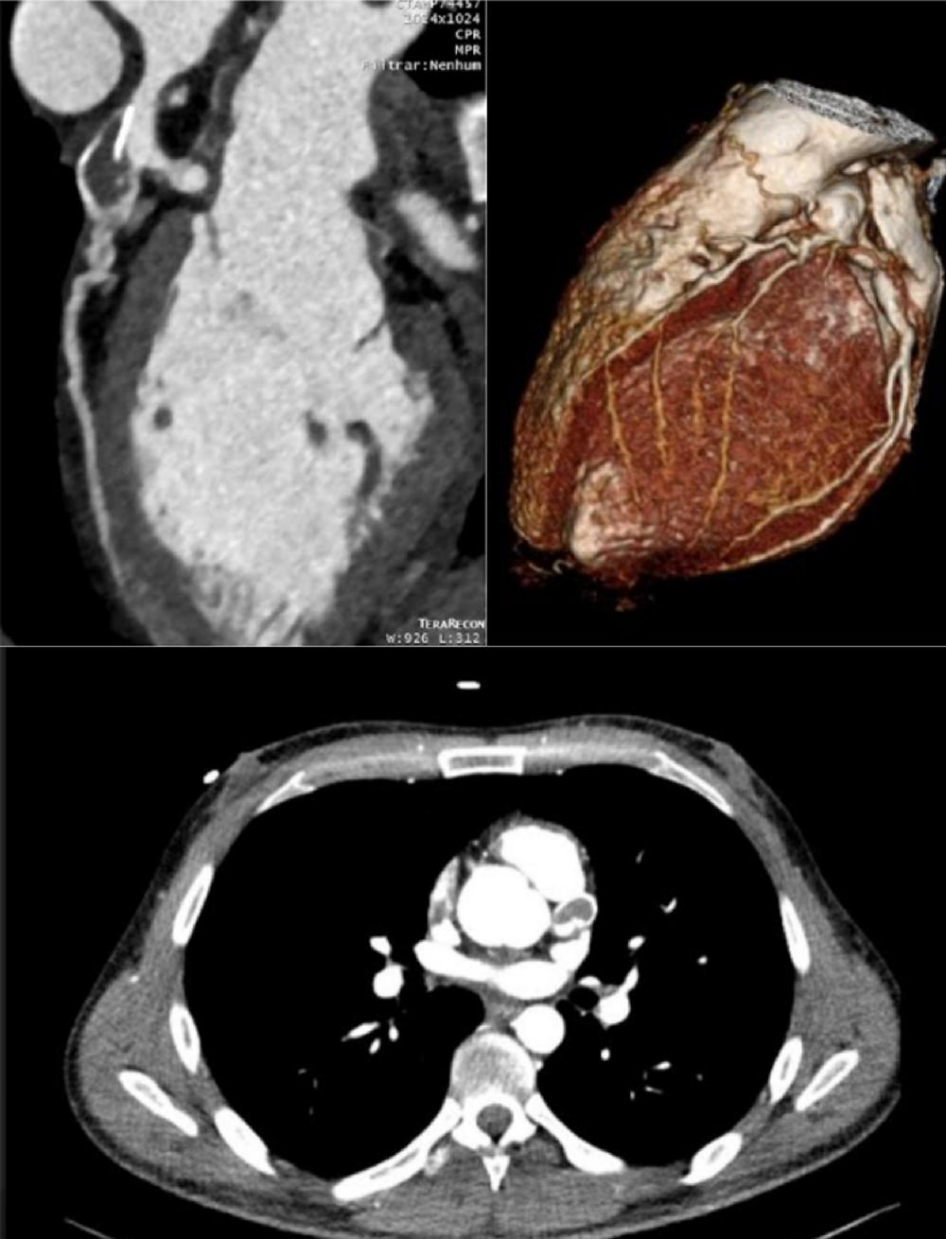
Computed tomography angiography—aneurysm involving the distal left main trunk and the ostial portion of the left anterior descending artery, with thrombosis causing subocclusive stenosis. Two sequential aneurysms are visible in the left circumflex artery, displaying parietal calcification without significant stenosis. The mid-segment of the right coronary artery reveals an ectatic segment with mural thrombosis, remaining patent with anterograde filling through collateral vessels.

Given the aneurysmal disease, the patient underwent a computed tomography angiography (CTA) (*Figure 2*) examination and was subsequently recommended for coronary artery bypass grafting (CABG). The coronary CTA scan revealed a significant aneurysm involving the distal left main trunk and the ostial portion of the LAD, with thrombosis causing subocclusive stenosis. Additionally, two sequential aneurysms were found in the LCx, with parietal calcification but no significant stenoses. The mid-segment RCA showed an ecstatic segment with mural thrombosis but remained patent, allowing for anterograde filling through collateral vessels.

A CTA scan was conducted on the cranial, supra-aortic, and abdominopelvic vessels, demonstrating no aneurysms. Therefore, the CABG was promptly performed on the fifth day of admission. The surgical procedure comprised the following sequential anastomoses left internal mammary artery to the LAD artery, right internal mammary artery (RIMA) to the first obtuse marginal artery (OM1), and RIMA to the interventricular posterior artery. Showing a positive evolution, the patient was discharged from the intensive care unit and transferred to a general ward 2 days after surgery with dual anti-platelet and statin therapy (aspirin 100 mg/day, ticagrelor 180 mg/day, rosuvastatin 200 mg/day) and was discharged from the hospital one week later.

The patient is under follow-up in the cardiology consultation and, six months post-infarction, reports no cardiovascular symptoms, classified as NYHA (New York Heart Association) class I and CCS (Canadian Cardiovascular Society) class 0. Laboratory tests are unremarkable, and the echocardiogram shows preserved global systolic function with no kinetic changes.

## Discussion

Kawasaki disease is diagnosed clinically as there is no specific test for its identification. However, laboratory and echocardiographic findings, such as elevated erythrocyte sedimentation rate and C-reactive protein levels, hyponatraemia, hypoalbuminaemia, and the presence of coronary aneurysms, can aid in evaluating suspected cases and distinguishing KD from other conditions.^5^ In certain instances, patients may not meet the classic criteria for KD and are thus classified as having IKD disease so clinical diagnosis and treatment are easily delayed. The risk of CAA is greater in incomplete Kawasaki.

In this clinical case, the patient was not diagnosed with KD due to advanced age, and weak clinical presentation. The condition was instead diagnosed as Still’s disease, even though the patient presented with elevated inflammatory markers, hyponatraemia, hypoproteinaemia, and a prolonged febrile syndrome, and was not treated with IVIG. The criteria for IKD disease have been broadened to facilitate early IVIG use, even for patients who do not fully meet diagnostic criteria. This approach prioritizes preventing undertreatment over potential overtreatment risks, as IVIG can effectively shorten the disease duration and mitigate complications like aneurysms.

The standard treatment for KD involves administering a single high dose of IVIG (2 g/kg) alongside aspirin until normal echocardiogram on follow-up (minimum 6 weeks) (3–5 mg/kg/day orally). Initiating this treatment within the first 10 days of illness significantly reduces the likelihood of coronary artery abnormalities (CAA), with the best outcomes in preventing cardiac complications observed when treatment begins before Day 7.^6^ Cases that don’t respond to IVIG—characterized by persistent or recurrent fever within 36 h to two weeks post-treatment—have a higher chance of developing complications. Research, including meta-analyses, indicates that adding high-dose steroids to IVIG in high-risk patients can further reduce the incidence of CAA by enhancing the anti-inflammatory response.^1^

Coronary artery aneurysms are usually an incidental finding on coronary angiogram; most are asymptomatic, although patients can present with angina pectoris, fistula formation, or sudden death.^7^ The occurrence of a coronary artery aneurysm in this patient prompted consideration of vasculitis, particularly KD and Takayasu’s arteritis. Kawasaki disease predominantly affects the coronary arteries, unlike Takayasu’s arteritis, which typically impacts the aorta and its major branches. The patient’s CT scan revealed a normal aorta and upper thoracic branches, making Takayasu’s arteritis less likely. Atherosclerosis is accountable for >90% of CAAs in adults, however, the patient was young and had no cardiovascular risk factors, with an LDL level of 55 mg/dL.

The management options for coronary aneurysms are still not well-defined and pose a therapeutic challenge to interventional cardiologists.^8^ Surgical intervention is indicated for symptomatic patients with obstructive coronary artery disease or evidence of embolization causing myocardial ischaemia. It is also recommended for patients with coronary artery aneurysms at risk of rupture. The CABG in this patient proceeded without complications, and the patient was discharged early with dual antiplatelet therapy and statin and blood pressure control as this patient should have their cardiac risk factors aggressively dealt with, whether they have obstructive coronary artery disease.

## Conclusion

Diagnosing KD, particularly its incomplete form, can be challenging due to overlapping symptoms with infections or rheumatic diseases. Children with unexplained fever should be closely monitored, as early recognition of KD is crucial to prevent complications. Coronary artery aneurysms are rare and often discovered incidentally during coronary angiography. While most CAAs are due to atherosclerosis, they can also be congenital or secondary to inflammatory or connective tissue disorders, notably KD. Some CAAs may become blocked, leading to severe complications like heart attacks, arrhythmias, or sudden death. Management may include surgical, percutaneous, or medical interventions, but determining the optimal treatment remains debated.

## Data Availability

Non-identifiable data underlying this article will be shared on reasonable request to the corresponding author.

## Lead author biography

Mariana Caetano Coelho, currently completing cardiology residency in Portugal, with a special interest in clinical cardiology and interventional cardiology.

## Notes

**Consent:** We obtained the patient’s consent in compliance with COPE guidelines.

**Funding:** None declared.

## References

[1] McCrindle BW, Rowley AH, Newburger JW, Burns JC, Bolger AF, Gewitz M, et al Diagnosis, treatment, and long-term management of Kawasaki disease: a scientific statement for health professionals from the American Heart Association. Circulation 2017;135:e927–e999. 28356445 10.1161/CIR.0000000000000484

[2] Saguil A, Fargo M, Grogan S. Diagnosis and management of Kawasaki disease. Am Fam Physician 2015;91:365–371. 25822554

[3] Newburger JW, Takahashi M, Gerber MA, Gewitz MH, Tani LY, Burns JC, et al Diagnosis, treatment, and long-term management of Kawasaki disease: a statement for health professionals from the Committee on Rheumatic Fever, Endocarditis and Kawasaki Disease, Council on Cardiovascular Disease in the Young, American Heart Association. Circulation 2004;110:2747–2771. 15505111 10.1161/01.CIR.0000145143.19711.78

[4] Holmes DR Jr, Vlietstra RE, Mock MB, Reeder GS, Smith HC, Bove AA, et al Angiographic changes produced by percutaneous transluminal coronary angioplasty. Am J Cardiol 1983;51:676–683. 6219567 10.1016/s0002-9149(83)80114-3

[5] Vervoort D, Donné M, Van Gysel D. Pitfalls in the diagnosis and management of Kawasaki disease: an update for the pediatric dermatologist. Pediatr Dermatol 2018;35:743–747. 30338568 10.1111/pde.13620

[6] Bal AK, Prasad D, Umali Pamintuan MA, Mammen-Prasad E, Petrova A. Timing of intravenous immunoglobulin treatment and risk of coronary artery abnormalities in children with Kawasaki disease. Pediatr Neonatol 2014;55:387–392. 24636168 10.1016/j.pedneo.2013.11.007

[7] Jha NK, Ouda HZ, Khan JA, Eising GP, Augustin N. Giant right coronary artery aneurysm—case report and literature review. J Cardiothorac Surg 2009;4:18. 19405985 10.1186/1749-8090-4-18 PMC2688487

[8] Bajaj S, Parikh R, Hamdan A, Bikkina M. Covered-stent treatment of coronary aneurysm after drug-eluting stent placement: case report and literature review. Tex Heart Inst J 2010;37:449–454. 20844620 PMC2929875

